# Systematic bacteriophage selection for the lysis of multiple *Pseudomonas aeruginosa* strains

**DOI:** 10.3389/fcimb.2025.1597009

**Published:** 2025-05-23

**Authors:** Finja Rieper, Johannes Wittmann, Boyke Bunk, Cathrin Spröer, Melanie Häfner, Christian Willy, Mathias Müsken, Holger Ziehr, Imke H.E. Korf, Dieter Jahn

**Affiliations:** ^1^ Pharmaceutical Biotechnology, Fraunhofer Institute for Toxicology and Experimental Medicine (ITEM), Braunschweig, Germany; ^2^ Institute of Microbiology, Braunschweig University of Technology, Braunschweig, Germany; ^3^ Leibniz Institute DSMZ-German Collection of Microorganisms and Cell Cultures GmbH (DSMZ), Braunschweig, Germany; ^4^ Department Trauma & Orthopedic Surgery, Septic & Reconstructive Surgery, Research and Treatment Center Septic Defect Wounds, Federal Armed Forces of Germany, Military Academic Hospital Berlin, Berlin, Germany; ^5^ Central Facility for Microscopy, Helmholtz Centre for Infection Research (HZI), Braunschweig, Germany; ^6^ Institute of Microbiology, Braunschweig Center of Systems Biology (BRICS), Braunschweig, Germany

**Keywords:** bacteriophages, *Pseudomonas aeruginosa*, phage susceptibility testing, antibiotic resistance, phage selection

## Abstract

*Pseudomonas aeruginosa* is an opportunistic pathogen causing severe infections of the lung, burn wounds and eyes. Due to its intrinsic high antibiotic resistance the bacterium is difficult to eradicate. A promising therapeutic option is the use of *P. aeruginosa*-specific bacteriophages. Thus, the implementation of a phage therapy requires their selection, production and systematic administration using multiple strains of the bacterial target. Here, we used 25 phages and tested their susceptibility on 141 different *P. aeruginosa* strains isolated from patients with different types of infection. Comparative host spectrum analyses were carried out using double agar overlay plaque assay (DPA) and planktonic killing assay (PKA), which resulted in 70% of the cases in the same host range. All phages were assigned to known phage genera, but some of the phages are new species. Isolated members of the genera *Pakpunavirus*, *Pbunavirus* (myoviruses), *Pawinskivirus*, *Elvirus* (myoviruses, jumbo phages), *Litunavirus* and *Bruynoghevirus* (podoviruses) demonstrated great therapeutic potential due to strong lysis behavior on diverse strains. Seven phages were excluded for therapeutic purposes due to genetic determinants that confer lysogenicity. Due to automation with lower time expenditure in execution and analysis, PKA has the higher potential for implementation in diagnostics. Finally, different combinations of phages were tested *in silico* with various *P. aeruginosa* strains. Highly efficient phage combinations eradicating multiple *P. aeruginosa* strains were found. Thus, a solid basis for the development of a broad host range phage therapy was laid.

## 1 Introduction

*P. aeruginosa* is a Gram-negative rod-shaped bacterium that is ubiquitous found in the environment. But, it also causes infectious diseases that often lead to long and expensive antibiotic treatments due to a large number of intrinsic antibiotic resistance and virulence factors (Aloush et al., 2006; Azam and Khan, 2019; Horcajada et al., 2019; Hilliam et al., 2020; Nazarov, 2022; Qin et al., 2022). In some cases, none of the eight antibiotic classes, including the reserve antibiotic class of carbapenems, commonly employed for *P. aeruginosa* infection treatment are effective anymore (Bassetti et al., 2018; Miller and Arias, 2024). For this reason *P. aeruginosa* is classified as high-risk pathogen on the World Health Organization’s global priority list for antibiotic-resistant bacteria (https://www.who.int/publications/i/item/9789240093461, accessed on 19 Nov 2024 (Miller and Arias, 2024; World Health Organization, 2024). Common sites of infection with *P. aeruginosa* in the human body are the respiratory tract, urinary tract, burn wounds, or the eyes (Poggio et al., 1989; Schein et al., 1989; Cheng et al., 1999; Serra et al., 2015; Prevaldi et al., 2016; La Rosa-Carrillo et al., 2022). Particularly in patients with predispositions like cystic fibrosis, bronchiectasis, chronic obstructive pulmonary disease (COPD), vascular diseases, diabetes, or immunocompromised patients there is a high risk of infection with *P. aeruginosa* (Prevaldi et al., 2016; La Rosa-Carrillo et al., 2022). Additionally, infections in difficult to reach sites in the body i. e. (eye background, sputum of cystic fibrosis lung) in combination with an intensive biofilm formation lead to poor pharmacokinetic distribution of applied antibiotics.

Consequently, other forms of bactericidal treatments are urgently required. One long-time known alternative and supplement to antibiotics is the use of bacteriophages (phages), viruses that infect bacteria. Phages show different pharmacokinetics and pharmacodynamics compared to classical drugs, as they multiply and self-regulate at the site of infection (Payne et al., 2000; Danis-Wlodarczyk et al., 2021b).

Around 10^31^ virus particles are estimated to exist on earth (Mushegian, 2020). But only a small part of these viruses, 2,818 genera, 84 subgenera and 11,273 species, have been classified by the International Committee for Taxonomy of Viruses (ICTV) in 2023 (International Committee of Taxonomy of Viruses, 2023). Of the sequences that can be unambiguously assigned to *P. aeruginosa* phages so far, about 2,148 nucleotide sequences, with 275 reference sequences and 55 different already classified genera have been deposited in the NCBI virus data base (https://www.ncbi.nlm.nih.gov/labs/virus/vssi/#/ accessed on 25 Oct 2024 (Hatcher et al., 2017; Alipour-Khezri et al., 2024). Due to the ubiquitous nature of *P. aeruginosa*, corresponding phages are found in soil, aquatic habitats, sewage, but also in humans and animals (Garbe et al., 2011; Amgarten et al., 2017; Shi et al., 2020; Aghaee et al., 2021; Alkalay-Oren et al., 2022; Hashemi Shahraki et al., 2023). Most *P. aeruginosa* phages described so far, including jumbo phages, belong to the *Caudoviricetes* (dsDNA genome) (Lecoutere et al., 2009; Yuan and Gao, 2017). A small part belongs to the *Inoviridae* (ssDNA), *Fiersviridae* (ssRNA) or *Cystoviridae* (dsRNA) (Sepúlveda-Robles et al., 2012; Pires et al., 2015; NCBI Virus, 2023). Shortly after the discovery of the phages by Frederick Twort and Félix Hubert d´Hérelle in the beginning of the last century, the latter developed the first phage therapeutic approaches together with the Georgian microbiologist Georgi Eliava. He founded the Bacteriological Institute Tiflis, later on renamed to the George Eliava Research Institute of Bacteriophage (Eliava Institute), today one of the leading institutions for phage therapy, even though most Western countries lost interest in phage therapy research during the time of the discovery and development of antibiotics. One major target of their research was the treatment of cystic fibrosis patients with *P. aeruginosa* infections (Parfitt, 2005; Chanishvili et al., 2022). Today, *Pseudomonas* phages have already shown their therapeutic potential against *P. aeruginosa* in several *in vitro*, *in vivo* and compassionate case studies on acute and respiratory infections, bacteremia, and wound infections (Debarbieux et al., 2010; Waters et al., 2017; Arumugam et al., 2022; Silva et al., 2022; Onallah et al., 2023; Rappo et al., 2023; Alipour-Khezri et al., 2024). The phages used therapeutically belong to twelve different phage genera - *Pakpunavirus*, *Pbunavirus*, *Phikzvirus* and *Nankokuvirus* (morphotype myovirus); *Litunavirus*, *Bruynoghevirus*, *Paundecimvirus* and *Phikmvvirus* (morphotype podovirus); *Septimatrevirus* and *Nipunavirus* (morphotype siphovirus) and *Perrunavirus* and *Cystovirus* (morphotype enveloped, spherical or icosahedral virion) (Rose et al., 2014; Ferry et al., 2021; Mabrouk et al., 2022; Silva et al., 2022; Alipour-Khezri et al., 2024; Pirnay et al., 2024; Pye et al., 2024). In general, prior to the treatments, phages were checked for an obligatory lytic lifecycle and the absence of virulence factors, toxins or antibiotic resistance genes in their genomes (Alipour-Khezri et al., 2024; Pirnay et al., 2024). Two generally different strategies exist for the application of phage therapy for the treatment of *P. aeruginosa* infections. Firstly, the use of a pre-composed phage cocktail such as practiced in the PhagoBurn project (Jault et al., 2019), composed of 12 phages directed against *Pseudomonas* infections of burn wounds, the various cocktails from the Eliava Institute in Georgia (Chanishvili, 2012) and from BiomX Gaithersburg, MD, USA (Rappo et al., 2023). In most cases their composition is not publicly available. The commercial cocktail of BiomX BX004-A in combination with antibiotics provided good tolerability and a bacterial reduction of 1.42 log in a clinical phase 1b/2a study of cystic fibrosis patients (Rappo et al., 2023). Secondly, a magistral preparation, as used in Belgium, Israel or Germany as part of the PhagoFlow project (Onallah et al., 2023; Pirnay and Verbeken, 2023; Willy et al., 2023), enables a customized selection of phages for each patient, often employed in combination with antibiotics (Onallah et al., 2023; Pirnay and Verbeken, 2023). Clinical improvement was reported for 77.2% of 100 cases of *P. aeruginosa* infections. In 61.3% of the cases a complete eradication of the targeted bacteria was found (Onallah et al., 2023; Pirnay et al., 2024).

A crucial factor during the establishment of phage therapeutic approaches is the quantitative determination of the efficiency phage activity using a phage susceptibility test (PST). Classically, a double agar overlay plaque assay (DPA) is performed that visualizes phage infection as plaques (Kropinski et al., 2009). Variations of DPA include the use of only one phage concentration or different dilutions applied as spots or discs with phages (RPST) (Skusa et al., 2023). Alternatively, phage lysis behavior can also be studied in liquids using a planktonic killing assay (PKA). To date, no standard conditions, cut-offs and breakpoints for bacterial lysis in the PST have been defined to determine the efficacy of phage lysis, which would contribute to comparability between laboratories (Parmar et al., 2023; Yerushalmy et al., 2023). It is already known from previous studies that the DPA and PKA methods lead to different results. One advantage of PKA is that the phages can be analyzed not only individually but also in combinations, which allows the identification of synergistic and antagonistic effects (Haines et al., 2021; Steffan et al., 2022).

Nevertheless, a systematic collection of *P. aeruginosa* phages tested in various combination on a broad spectrum of *P. aeruginosa* strain isolated from different types of infection is missing to generate generally applicable knowledge for *P. aeruginosa* phage therapies. This is necessary to select safe and broad host spectrum phages for a clinical magistral application to individually treat as many patients as possible. Thus, in this project, 25 phages and 141 P. *aeruginosa* strains were isolated, characterized, sequenced and their interaction investigated. In the context of phage susceptibility, DPA was compared with serial dilutions as spots and PKA on a large phage-host panel to reveal the advantages and disadvantages of each of the method.

## 2 Materials and methods

### 2.1 Bacterial strains and growth conditions

*P. aeruginosa* strains BWKH001–133 were isolated by the military hospital in Berlin and Hamburg (Germany) between 2015 and 2018. A description of the corresponding infection type (wounds, skin, repository tract, urinary tract, rectal, tissue) is given in **Supplementary Data Sheet 1**. Additionally, *P. aeruginosa* MH16, MH19, MH27, MH38 and RN21 were isolated from the urinary tract (Tielen et al., 2011, 2014). Five *P. aeruginosa* strains from patients with cystic fibrosis, five strains from patients with chronic obstructive pulmonary disease (COPD56-COPD129) and 14 strains of bronchiectasis (Bron08-Bron76) patients were provided by B. Tümmler (Medical School Hannover MHH, Germany) (Hamed et al., 2023). The model strains PAO1 (DSM 19880) and PA14 (DSM 19882) were obtained from the German Collection of Microorganisms and Cell Cultures (DSMZ Braunschweig, Germany) (Rahme et al., 1995; Stover et al., 2000; Mathee, 2018). All *P. aeruginosa* strains were cultivated aerobically in LB veggie medium (10 g/L veggie peptone, 5 g/L veggie yeast extract, 10 g/L NaCl (all Merck, Darmstadt, Germany)) at 37°C. For growth on agar plates 1.5 g/L agar (Merck, Darmstadt, Germany) was added.

### 2.2 Antibiograms of the *P. aeruginosa* strains

Bacterial strains were tested for antibiotic susceptibility using the disc diffusion method. Discs according to EUCAST concentrations were ordered by Mast Diagnostica GmbH, Reinfeld/Germany (**Supplementary Data Sheet 2**). Briefly, freshly streaked bacteria from cryo culture (LB veggie agar, 37°C, 18 h) were suspended in 10 mL 0.85% NaCl (Merck, Darmstadt, Germany) solution to an OD_600nm_ of 0.25. A cotton swab was dipped once into the inoculum, squeezed, and streaked in three directions on Mueller Hinton agar plates (25 mL/dish, 38 g/L, Roth, Karlsruhe, Germany). Afterwards six antibiotic discs were placed per plate with a dispenser (Mast Diagnostica GmbH, Reinfeld, Germany). After an overnight growth (35°C, 17h ± 1h) the zone of inhibition was measured and compared to the EUCAST thresholds (European Committee on Antimicrobial Susceptibility Testing, 2022).

### 2.3 Phage isolation, purification, and propagation

For isolation of *P. aeruginosa* phages, enrichments from different environments in Germany, including clinical sewage, washing machines and garden compost were performed between 2007 and 2022 and detected by DPA as described by Kropinski et al. with minor changes (Kropinski et al., 2009). A list of the phages and their characteristic is given in **Supplementary Data Sheet 3**. Agar concentration in top agars for host range analyses was 0.5% (Sigma Aldrich, Darmstadt, Germany), while different top agar concentrations (0.3 to 0.7%) were applied for visualization of the individual phages (**Supplementary Data Sheet 3**). Clonal purification was performed by in least three consecutive rounds of single plaque picking and streaking out on double ager overlay plates. Plaque sizes were determined via ruler measurement. In general, phage lysates were prepared either in liquid form by infecting logarithmically growing *P. aeruginosa* cultures with phages at a multiplicity of infection (MOI) between 0.01 to 0.5 followed by an incubation at 37°C and 140 rpm until complete lysis or for 22 hours. Alternatively, top agar of double agar overlay plates were incubated with phages until semiconfluent lysis occurred, subsequently covered with SM buffer, and finally scraped off after incubation. After centrifugation (10,967 x g, 4°C, 10 min), the phage-containing supernatant was filtered through 0.2 µm syringe filters (cellulose acetate, Sartorius, Germany), quantified using DPA and stored at 4°C.

### 2.4 DNA isolation and determination of DNA concentration

For DNA isolation the “Phage DNA Isolation Kit” (Norgen, Thorold, Canada) was used following the manufacturer’s protocol and DNA was stored at 4°C. The concentration was determined using the Qubit^®^ dsDNA broad range and 1x dsDNA high-sensitive assay kit (Thermo Fisher Scientific, Waltham, USA) following the manufacturer’s instructions.

### 2.5 Library preparation and whole genome sequencing

The protocols for library preparation and whole genome sequencing for PacBio RSII and Illumina were described before (Korf et al., 2019). Other long read sequencing of phage genomes was performed with Nanopore technology using the protocol, “Ligation sequencing gDNA - SQK-LSK109 version: GDE_9063_v109_revAP_25May2022” (ONT, Oxford, United Kingdom), flongles version 9.4.1 with default parameters and basecaller Guppy version 7.1.4 with high-accuracy model.

### 2.6 Genome assembly, annotation and comparison

Either SPAdes version 3.12.0 (PacBio and Illumina) or flye version 2.9.3-b1794 (ONT and Illumina) was used for genome assembly, before long reads were trimmed with porechop version 0.2.4 (threshold of 10). Short reads were mapped using BWA short reads version 0.7.17.5 with default parameters and polished using polypolish version 0.5.0 (default parameters). All genomes, including those already published, were annotated using pharokka version 1.7.1 and compared with clinker version v0.0.28. Phylogenomic tree was performed with VICTOR (Meier-Kolthoff and Göker, 2017), visualized with iTOL (Letunic and Bork, 2024) and annotation was generated with table2itol (https://github.com/mgoeker/table2itol).

### 2.7 Morphological analysis via transmission electron microscopy

For TEM analysis, phages were prepared for analysis as previously described (Korf et al., 2019). Briefly, thin carbon support films were prepared by evaporating a carbon thread onto a freshly cleaved mica surface. Small pieces of mica were then cut, and phages were negatively stained with 2% (w/v) aqueous uranyl acetate, pH 5.0. The samples were examined at an acceleration voltage of 80 kV/120 kV in a Zeiss EM 910 or Zeiss Libra120 Plus transmission electron microscope (Carl Zeiss, Oberkochen, Germany). The dimensions of heads and tails was determined for 3–10 different phage particles using ITEM software (Olympus Soft Imaging Solutions, Münster, Germany). The head dimension was estimated on the basis of head length x head width. The phenotypic classification was done using the morphological criteria of Ackermann (Ackermann and Eisenstark, 1974; Ackermann, 2011).

### 2.8 Host range analysis by DPA (double agar overlay plaque assay)

The phage host range was determined by spotting serial dilutions (adjusted to 1E9 PFU/ml) on double agar plates containing 100 µL of a logarithmic culture of the potential host. After incubation for 18h ± 2h at 37°C, the plates were examined for lysis. The host was categorized as sensitive if individual plaques could be detected (lysis), or as insensitive if either no visible plaques (no lysis) or reduced growth was observed.

### 2.9 Host range analysis by PKA (planktonic killing assay)

A PKA was performed to analyze the host range of the phages in liquid bacterial culture. A culture was inoculated with an overnight culture to an OD_600nm_ of 0.05, allowed to grow above an OD_600nm_ of 0.1 and finally adjusted to an OD_600nm_ of 0.1. 200 µL adjusted culture were infected with 10 µL phage (2E8 PFU/mL) to reach an approximate MOI of 0.1. The optical density was measured in SpectraMax 250 (MWG-Biotech, Ebersberg, Germany) at 600 nm every 15 min for 24 h. The 96 well plate was mixed for 3 s before measurement. The host was classified as sensitive when the normalized area under curve (AUC_norm_) was < 0.8 (lysis) and insensitive when AUC_norm_ was ≥ 0.8 (no lysis).


$$AUC_{norm}=\frac{\;{\left[AUC\right]}_{0h}^{24h}with\;phage}{{\left[AUC\right]}_{0h}^{24h}without\;phage}$$

For all combinations that were classified as lytic, the time point of lysis was calculated. This value was set as the first time point, where AUC_norm_ was below 0.8.

### 2.10 Host range comparison of DPA and PKA

To compare DPA and PKA, corresponding results of their host spectra were merged in each possible combination. Resulting matches or deviations were grouped as follows: group 1 was “DPA and PKA tests resulted in no phage lysis”, group 2 was “DPA resulted reduced bacterial growth and PKA with no lysis”, group 3 was “DPA with lysis and PKA without lysis”, group 4 was “DPA with no lysis and PKA with lysis”, group 5 was “DPA with reduced growth and PKA with lysis”, and group 6 was “DPA and PKA with lysis”. The rows and columns of the heatmap were clustered using the distinct method in R software with resulting categories.

### 2.11 Determining the optimal phage combination

Optimal phage combinations based on host ranges identified by DPA and PKA were determined by Phage Cocktail Optimizer by Stephen T. Abedon (https://www.phage-therapy.org/calculators/cocktail_optimizer.html, accessed on 5 Jul 2024). “Lysis” determined as described above was rated as “positive” phage-host interaction.

### 2.12 MOI dependent planktonic killing assay and virulence index

The original bacterial host on which the phage was isolated was tested with different MOIs to determine the lysis behavior. The culture was inoculated from an overnight culture with OD_600nm_ of 0.05 and grown to an OD_600nm_ of 0.1. Serial dilutions of the culture at the time of infection were plated to determine the colony forming units (CFU) of the host *P. aeruginosa*. The phage was adjusted to 2E9 PFU/mL and serial diluted to 2E5 PFU/mL with LB veggie medium. 200 µL culture were infected with 10 µL phage dilutions in triplicates (final MOI between 1 to 0.0001). Optical density was measured using a SpectraMax 250 (MWG-Biotech, Ebersberg, Germany) every 15 min for 24 h. After incubation the triplicates were pooled and centrifuged (16,200 x g, 2 min, at room temperature) (Fresco 21, Thermo Scientific, Waltham, US). The phage titer t_24h_ was determined by DPA from corresponding supernatants. Local virulence index (vi) was assessed as previously described (Storms et al., 2020). The beginning of the stationary phase was defined as the first local maximum with a threshold value of 5:


$$v_{i}=1-\frac{\;{\left[AUC\right]}_{0h}^{stationary\;phase}with\;phage}{\;{\left[AUC\right]}_{0h}^{stationary\;phase}without\;phage}$$

The global virulence index (vp) was calculated as the quotient of the area under the virulence curve (A_p_) divided by the theoretical maximum area under the virulence curve (A_max_) (Storms et al., 2020):


$$v_{p}=\frac{A_{p}}{A_{max}}$$

### 2.13 Ranking score

For the final ranking phages received score for the categories DPA, PKA, virulence index (vp) and safety according to **Supplementary Data Sheet 4**.

## 3 Result

### 3.1 Isolation and initial characterization of 25 *P. aeruginosa* phages

**Figure 1:**
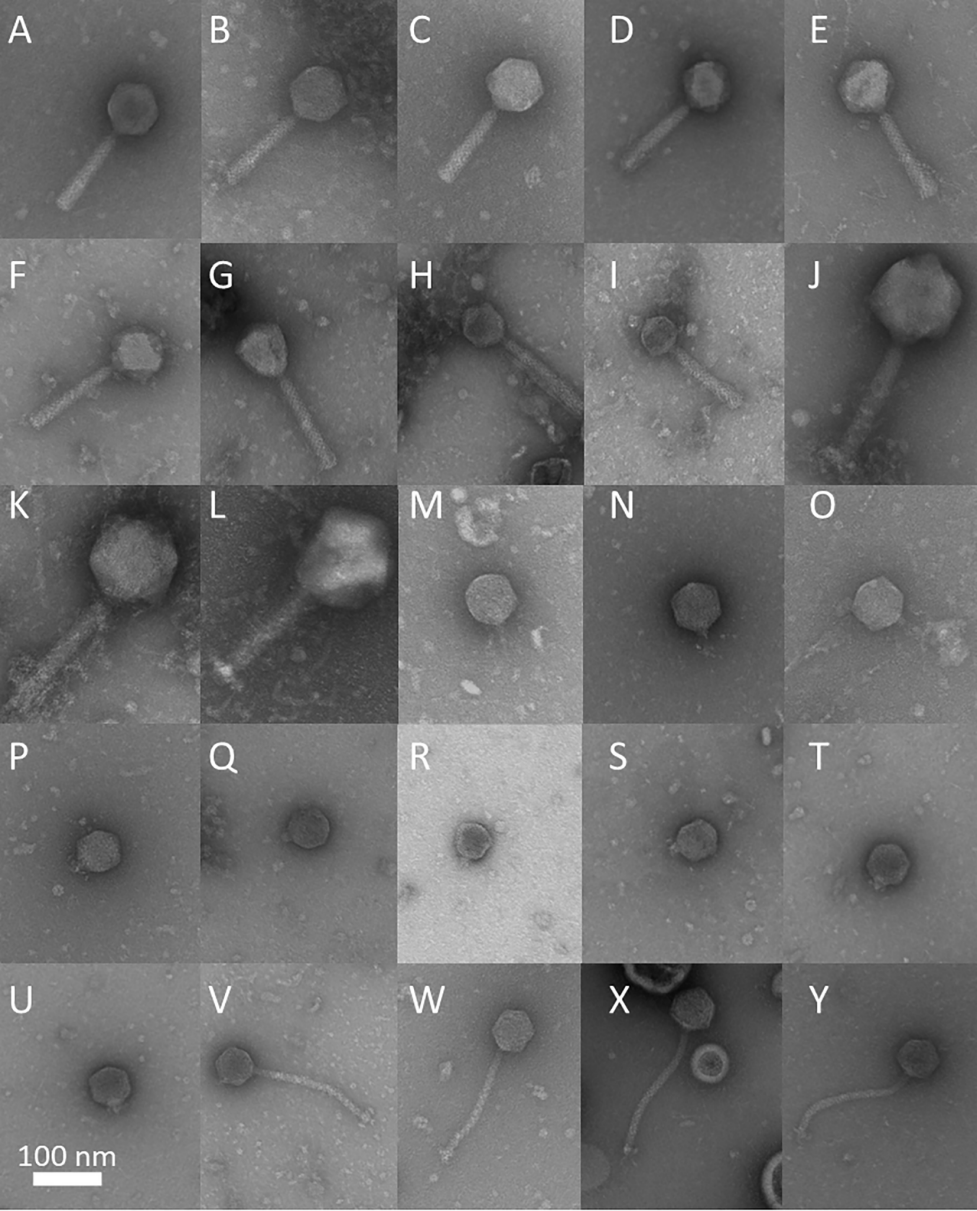
Electron micrographs of isolated phages with the morphotype myovirus [JG004 **(A)**, HHBS9_1 **(B)**, HHB18_1 **(C)**, PTLAW1 **(D)**, HHBS12_2 **(E)**, HHBS42_2 **(F)**, HHBS51_1 **(G)**, Komp_PAO1_1 **(H)**, BIBS67 **(I)**, HHBS47_1 **(J)**, HHBS8_1 **(K)**, HHBS36_1 **(L)**], and morphotype podovirus [BWKH3_L8_1 **(M)**, BWKH3_R8_1A **(N)**, HHBS9_2 **(O)**, HHBS10_2 **(P)**, HHBS55_2 **(Q)**, HHBS14_1 **(R)**, Flu_PA14_3 **(S)**, Flu_PA14_4 **(T)**, Komp_PA14_gP **(U)**] and morphotype siphovirus (HHBS29_1 **(V)**, 22043_B8_1 **(W)**, Tom33 **(X)**, Komp_PA14_H **(Y)**. Bar represents 100 nm.

**Figure 2:**
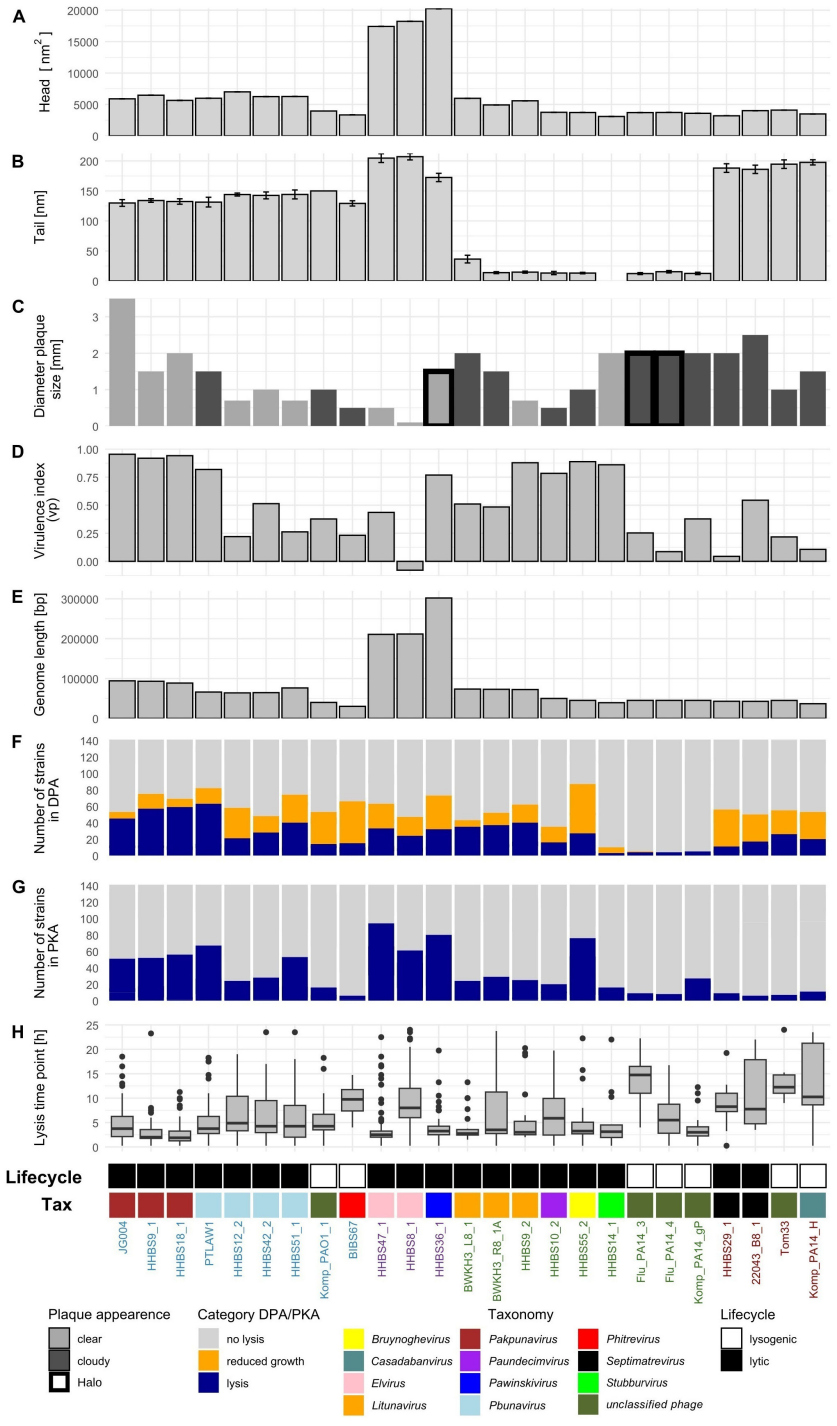
The diversity of the phages was assessed based on ten parameters. For all parameters phages are grouped based on morphotype (colored names at the bottom: myo- (blue) – jumbo phages (violet), podo- (green) or siphovirus (brown)) and taxonomy. Lysogenic phages are indicated in white, strictly lytic phages in black. Head size (head length x head width), tail length, plaque appearance, genome size, as well as virulence index (vp) are shown as barplots **(A-E)**. Distribution of lysis behavior in DPA and PKA resulting in either no lysis, reduced growth or lysis are displayed in **(F, G)**. Boxplots represent the time of lysis in PKA (first time normalized AUC was < 0.8) for all strains **(H)**.

In order to systematically test multiple *P. aeruginosa* strains isolated from highly different infections corresponding phages were selected as a first step. For this purpose, phages were isolated from wastewater (partly with clinical proximity), garden compost and washing machines between 2004 and 2022 from locations in northern Germany. After isolation and propagation, phages were systematically characterized for their head, tail, plaque size, halo formation, plaque appearance, genome size, number of genes, GC content, virulence index and lysis behavior as shown in **Figures 1**, **2** and **Supplementary Data Sheet 1** (complete dataset).

### 3.2 Phage morphology

Using transmission electron microscopy (TEM), the 25 phages were classified as twelve myoviruses including three jumbo phages, four siphoviruses and nine podoviruses (**Figure 1**). All phages had a symmetrical hexagonal head and were categorized in A1 (myoviruses), B1 (siphoviruses) and C1 (podoviruses) according to Ackermann and Eisenstark, 1974. Prolonged heads were not observed. When analyzing phage dimensions, the myovirus morphotype showed the greatest diversity (myoviruses: mean head height 93 ± 29 nm; mean head width 87 ± 25 nm; mean tail length 151 ± 26 nm, podoviruses: 67 ± 8 nm, 65 ± 6 nm; 16 ± 8 nm and siphoviruses 62 ± 4 nm, 59 ± 3 nm; 192 ± 5 nm (**Figures 2A, B**). The phages HHBS36_1 (*Pawinskivirus*), HHBS8_1 and HHBS47_1 (both *Elviruses*) are representatives of the jumbo phages with head heights ranging from 139 to 147 nm. This means that they are not only larger in terms of their genome (see Chapter 3.5.1), but also in terms of their head and tail.

### 3.3 Plaque morphology and halo formation of the isolated phages

The diversity of phages can also be illustrated by their plaque morphology (**Figure 2C**, **Supplementary Figure 1**). DPA was performed with different concentrations of top agar (**Supplementary Data Sheet 3**). Plaque size ranged between 0.1 mm at 0.3% top agar concentration for HHBS8_1 (*Elvirus)* to 3.5 mm at 0.7% top agar concentration for JG004 (*Pakpunavirus)*. Most myoviruses formed clear plaques, whereas the plaques of podoviruses and siphoviruses tended to be turbid. All temperate phages (**Supplementary Data Sheet 3**) produced turbid plaques. Halo structures could be found through all morphotypes. However, it should be noted that only 16% of all phages studied produced halos.

### 3.4 Lysis behavior of isolated phages

The actual virulence and efficacy of lysis were assessed in 96-well format using different MOIs and calculating the virulence index (vp) to compare the phages for later therapeutic potential (**Figure 2D**). The host bacteria used were the same as those used for the isolation. A high virulence index is characterized by rapid and complete lysis. Some phages of the morphotype myovirus (vp mean 0.58 ± 0.31) and podovirus (vp mean 0.57 ± 0.28) lysed in a short period and had a high virulence index of e. g. 0.95 - JG004 (*Pakpunavirus*) or 0.88 - HHBS9_2 (*Litunavirus*). On the other hand, there were also phages from the same morphotype that had a very low virulence index of 0.23 - BIBS67 (*Phitrevirus*) or 0.09 – Flu_PA14_4 (*unclassified phage*). For HHBS29_1 (*Septimatrevirus* – morphotype siphovirus) lysis was only observed at high MOI (1 and 0.1) so the virulence index was correspondingly low (vp 0.04). Two myoviruses JG004 and HHBS9_1 (both *Pakpunavirus*) underwent a second lysis after regrowth (**Supplementary Figure 2A**).

### 3.5 Genome of the isolated phages

#### 3.5.1 General genetic properties

Phage sequencing was performed using PacBio or ONT (long reads) and Illumina (short reads) technologies for taxonomic classification and to exclude unwanted genes. Phage genome size ranged from 30,180 bp (BIBS67 - *Phitrevirus*) to 302,046 bp (HHBS36_1 – *Pawinskivirus*) (**Figure 2E**). Phages of the jumbo phage genera *Elvirus* and *Pawinskivirus* reached the upper limit in both physical and genome size (mean 246,208 bp). Other myoviruses also tended to have slightly larger genomes (mean 68,619 bp) than podoviruses (56,496 bp) and siphoviruses (41,912 bp). The GC content was between 44% and 64%, while all temperate phages had higher GC content (mean 61%) then lytic phages (mean 52%).

#### 3.5.2 Phylogenetic tree of the isolated phages

**Figure 3:**
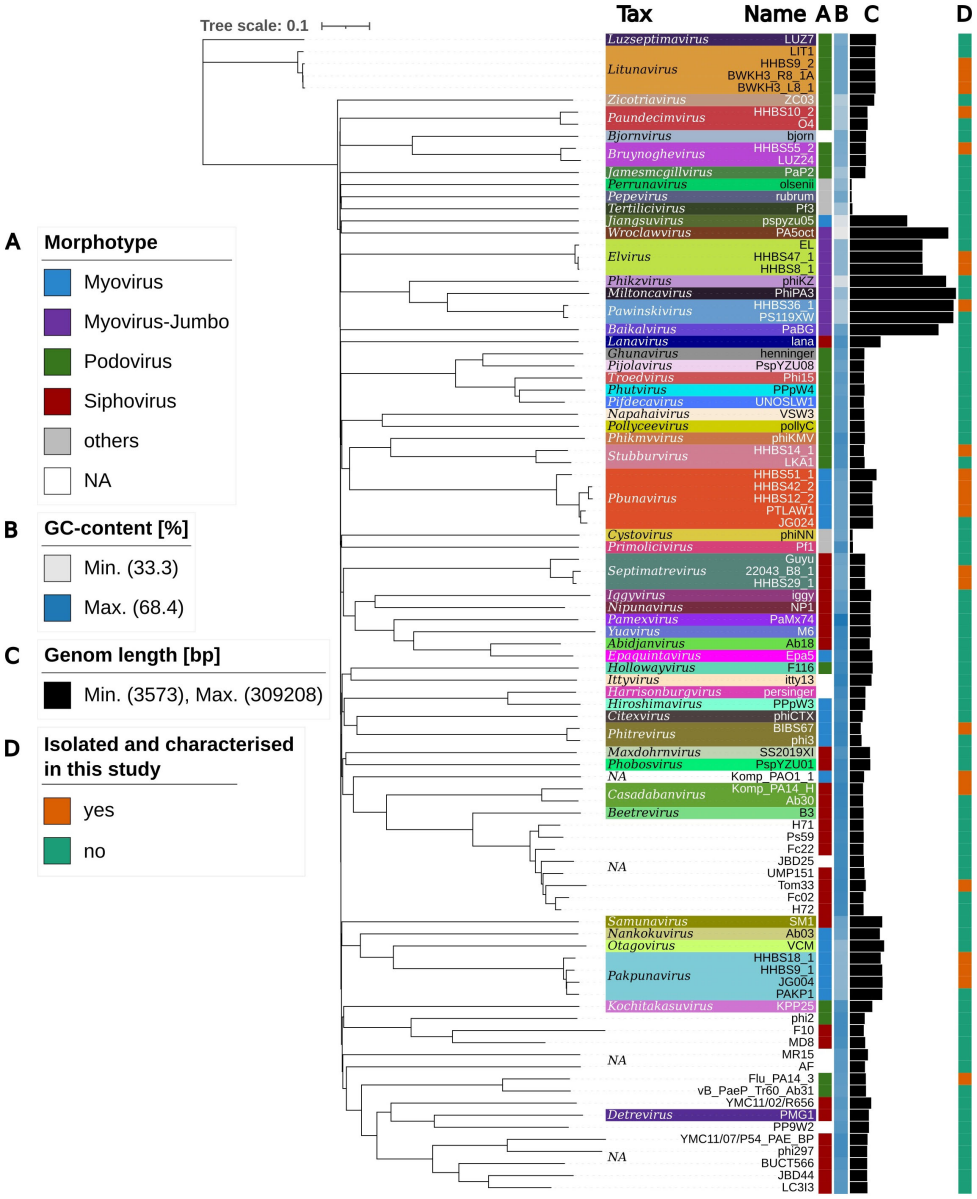
Phylogenomic tree of *P. aeruginosa* phages created with VICTOR and visualized with iTOL. At least one phage was selected from each *P. aeruginosa* phage genus uniquely colored (Tax). Morphotype and GC-content of phage are shown as color bars **(A, B)**. Genome size is displayed as bar chart **(C)**. All phages isolated and characterized in this study are labeled in orange **(D)**.

The diversity of *P. aeruginosa* phages can also be demonstrated by examining their genome. At the time of the study, 18,673 reference genomes were deposited in the NCBI Virus Database (https://www.ncbi.nlm.nih.gov/labs/virus/vssi/#/ accessed on April 2nd, 2025). If all phage genomes whose host does not contain the search terms “*Pseudomonas aeruginosa*”, “*Pseudomonas*” or “*Pseudomonas* sp.” were removed, 275 reference genomes remained. At least one genome from each *P. aeruginosa* phage genus (complete reference sequence deposited in NCBI Virus Database) was selected and the relationships were presented in a phylogenetic tree (**Figure 3**). The distribution of morphotypes is very diverse, as it is spread across different clusters. While *Pakpunavirus* and *Pbunavius* are clearly separated, the jumbo phages all cluster together and have a relatively low GC content. Members of *Bruynoghevirus*, *Paundecimvirus* and *Litunavirus* share only individual genes with transcriptional regulatory or metabolic function. This makes them more closely related to each other than to *Stubburvirus* (e.g. HHBS14_1) or unclassified podoviruses (e.g. Flu_PA14_3). The unclassified phages including Tom33 form a very distinct cluster, most of which perform a lysogenic cycle and have a relative high GC content (Carballo-Ontiveros et al., 2020). But even within a genus, the phages were quite different, as only a nucleotide identity of 70% (coverage * identity) is required (Turner et al., 2021). Twelve out of 55 P. *aeruginosa* phage genera were covered by the phage panel characterized in this study. Some genera have already been described frequently and have many reference species. For example, 38 reference species are known for *Pakpunavirus*, 37 for *Pbunavirus*, 13 for *Litunavirus* and 20 for *Septimatrevirus*, while only one reference phage exists for relatively new species such as *Elvirus* and *Pawinskivirus*.

However, despite the high sequence identity, the genome of phage species within these common genera sometimes differs considerably. The phages within *Litunavirus* and *Pakpunavirus* are more conserved than *Pbunavirus*, *Bruynoghevirus* and *Septimatrevirus*. In all genera, highly conserved genes (core genome) belong to proteins related to phage head and packaging, DNA, RNA and nucleotide metabolism (**Supplementary Figures 3**-**8**). When all phages are aligned with dnaapler at the same starting point (*terL*), the greatest variability is seen in proteins of the tail structures, such as the tail fiber protein, and at the end of the genome, where many small, hypothetical proteins are located. It is noteworthy that HHBS51_1 exhibits an insertion of 10 kb, which is not observed in other *Pbunavirus*. The insertion includes an endolysin, DNA primase and many small proteins of unknown function, but no genes that cause a lysogenic life cycle (**Supplementary Figure 3**).

### 3.6 Isolation and antibiotic sensitivity of 141 *P. aeruginosa* strains

Most *P. aeruginosa* isolates were isolated in the military hospitals in Berlin and Hamburg as well as at the Medical School in Hannover (MHH) in Germany from various types of infection, resulting in great diversity in terms of isolation time, geographical origin and indication (**Supplementary Data Sheet 1**). A detailed characterization of these strains will be subject of a different investigation. The susceptibility of *P. aeruginosa* isolates to different antibiotics (**Supplementary Figure 9**) was tested to compare susceptibility to antibiotics with susceptibility to phages. Twelve antibiotics including ceftazidime*, ceftazidime/avibactam, ceftolozane/tazobactam, cefiderocol (cephalosporins), piperacillin*, piperacillin/tazobactam (penicillins), imipenem*, meropenem* (carbapenems), azetreonam (monobactam), and ciprofloxacin* (fluoroquinolone), and some other antibiotics like amikacin and tobramycin (aminoglycosides) which are used as standard or reserved therapy for *P. aeruginosa* infections were chosen. All lead substances of antibiotic classes (underlined) were included (**Supplementary Data Sheet 2**). According to Robert-Koch-Institute in Germany, a distinction was made between 3 MRGN being resistant to three and 4 MRGN being resistant to four antibiotic classes (Kommission für Krankenhaushygiene und Infektionsprävention, 2019). Based on the antibiogram, 39% 3 MRGN (55/141 strains) and 61% 4 MRGN (86/141 strains) were identified (meropenem used as lead substance). Many wound isolates were only susceptible to cefiderocol, a last resort antibiotic. However, *P. aeruginosa* strains BWKH001, BWKH038 (both wound isolates) and Bron11 (respiratory tract) were even resistant to cefiderocol.

### 3.7 Host range of the phages tested with the 141 *P. aeruginosa* strains

**Figure 4:**
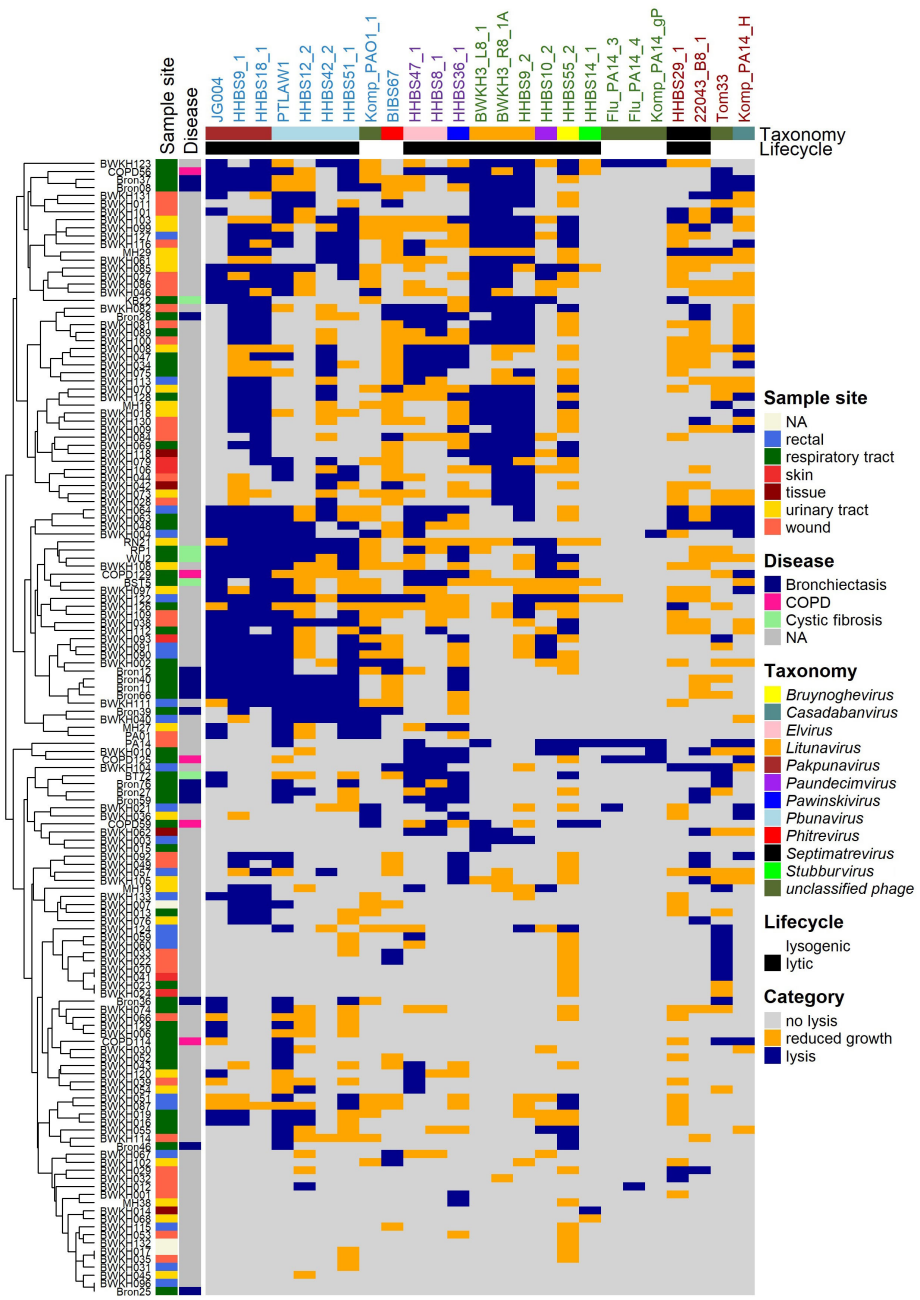
Host range analysis by DPA (top agar 0.5%) of 25 phages. Interaction is classified into “no lysis” (grey), “reduced growth” (orange) and “lysis” (blue). The used phages are colored based on their morphotype (colored names at the top: myo- (blue) – jumbo phages (violet), podo- (green) or siphovirus (brown)), taxonomic classification and lifestyle. Analyses was performed using 142 clinical isolates of *P. aeruginosa* sampled from different habitats and patients with different diseases (colored left). Clustering of rows is performed by distinct method of ComplexHeatmap package in R.

**Figure 5:**
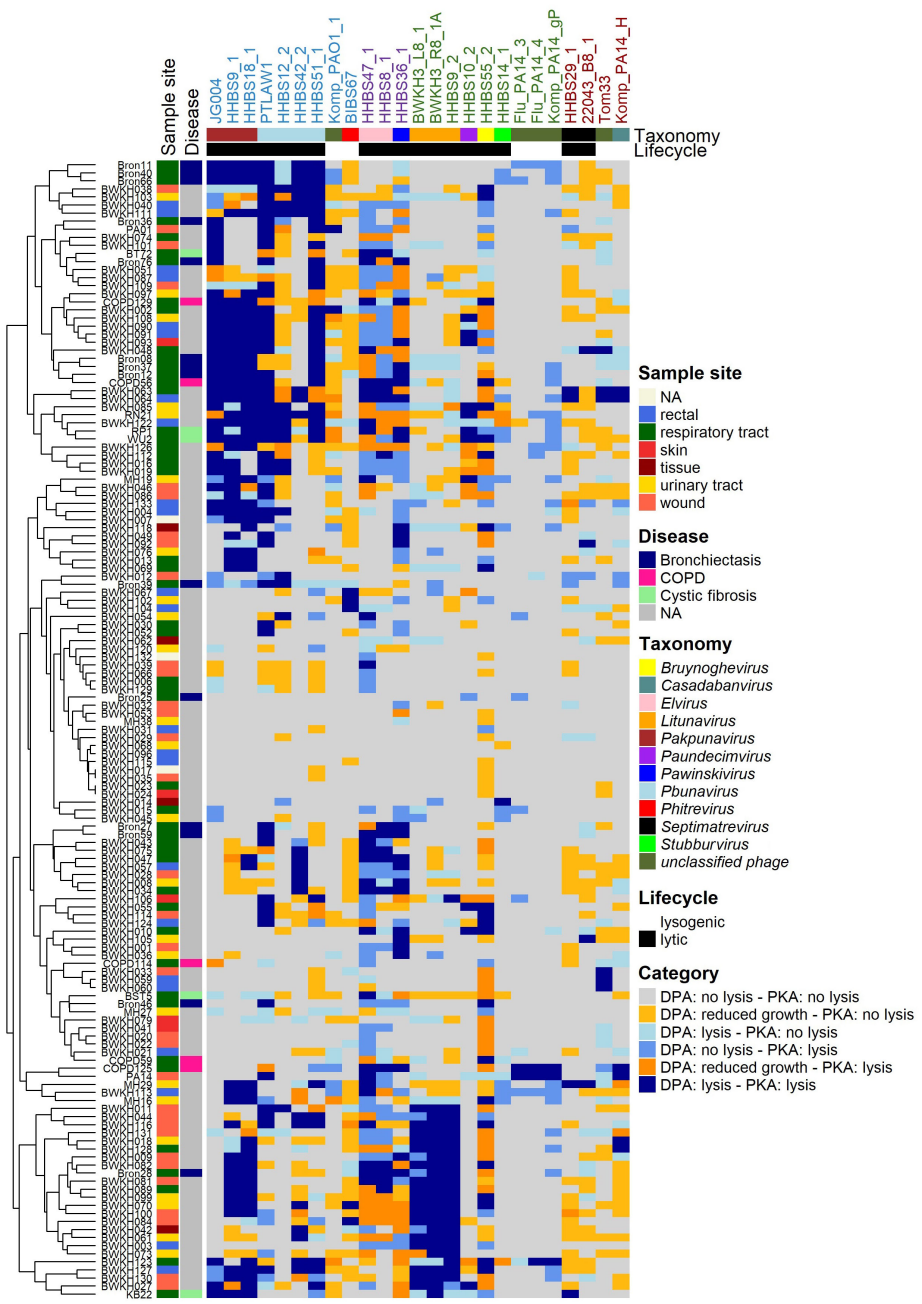
Comparison of host spectra determined by DPA and PKA respectively. Phages are colored based on their morphotype (colored names at the top: myo- (blue) – jumbo phages (violet), podo- (green) or siphovirus (brown)), genus and lifecycle (top) and the *P. aeruginosa* strains are shown with the sample site (left). DPA was performed using 0.5% top agar concentration and serial dilutions of phage lysates and classified as “no lysis”, “reduced growth” and “lysis”. PKA was performed in LB veggie medium at MOI 0.1. The growth curves of PKA are integrated for 24h and normalized against the control without phage. Normalized AUC ≥ 0.8 is classified as “no lysis” and normalized AUC < 0.8 as “lysis”. Both results are combined with the expressions DPA: lysis - PKA lysis in dark blue, DPA: reduced growth - PKA lysis in dark orange; DPA: no lysis - PKA lysis in mid blue; DPA: lysis - PKA no lysis in light orange; DPA: reduced growth - PKA no lysis in light blue and DPA: no lysis - PKA no lysis in grey. Heatmap is clustered with average methods of ComplexHeatmap (R) in rows and columns.

A classical host range analysis (DPA) was performed with the diverse phage panel (**Figures 2F**, **4**). *Pbunavirus* and *Pakpunavirus* had a broad host range (15 – 45%) and clustered close together although they are not genetically related (**Figure 3**). PTLAW1 (*Pakpunavirus*) had the highest coverage with 45% (63/141 strains). HHBS36_1 (*Pawinskivirus)*, HHBS47_1 and HHBS8_1 (both *Elviruses)* had a medium coverage of 21% (mean 30/142 strains), but phage-host interactions were often categorized as reduced growth, in 22% of interactions. Among the myoviruses, BIBS67, a temperate *Phitrevirus*, had a narrow host spectrum with a coverage of 11% (15/141 strains). In comparison to *Pakpunavirus*, the *Litunavirus* (podoviruses) grouped together more strongly and lysed other strains (see **Figures 4**, **5**). Twelve out of 33 wound and seven out of 24 urinary tract isolates were covered by *Litunavirus* with at least one phage. Strikingly, the *Bruynoghevirus* HHBS55_2 showed reduced growth for a particularly large number of strains 43% (60/141 strains), whereas plaques were only determined for 19% of the strains (27/141 strains). In contrast, HHBS14_1 (*Stubburvirus*) was very specific and covered 2% of the tested strains (3/141 strains). Similarly, Flu_PA14_3, Flu_PA14_4 and Komp_PA14_H (*unclassified phage*) covered 3 - 4% of the strains. The two *Septimatrevirus* (siphoviruses) were quite different from each other. HHBS29_1 showed lytic behavior on 8% of the strains (11/141 strains), some of which were cystic fibrosis isolates, and 22043_B8_1 lysed 12% of the strains (17/141 strains) (see **Figures 4**, **5**). Both phages tend to have many phage-host interactions that we classified as reduced growth. No plaques were detected in about 9% of the strains (13/141 strains) from different sampled sites. 10% (14/141 strains) of *P. aeruginosa* strains were only covered by one phage, showing visible plaques. On the other hand, there were also 10% (14/141 strains) of *P. aeruginosa* strains that could be covered by ten or more of the phages tested. Interestingly, the susceptibility of the *P. aeruginosa* strains against phages tended to correlate positively with the susceptibility of antibiotics (**Supplementary Figure 10**). Lytic behavior of phages could not be shown for *P. aeruginosa* strains BWKH17 (wound), BWKH23 (respiratory tract), BWKH24 (skin), BWKH35 (wound), BWKH68 (urinary tract), BWKH96 (rectal swab) or BWKH115 (rectal swab) with any method. All of them are highly resistant to antibiotics.

### 3.8 Comparison of the PKA and DPA methods

However, it is not yet clear which is the best *in vitro* method for determining the phage susceptibility that best predicts *in vivo* efficacy. Therefore, we determined the host range using both PKA and DPA method and systematically compared the results in order to contrast the advantages, disadvantages and limitations of both methods. The comparison of both methods revealed a correlation of 70%, of which 13% is attributable to a lytic interaction (dark green) and 57% to a non-lytic interaction (light green) (**Supplementary Figure 11A**). A high correlation of lytic and non-lytic interaction can be observed particularly in the genera *Pbunavirus*, *Pakpunavirus* and *Litunavirus* (**Supplementary Figure 11B**). At the same time, the PKA method tended to identify more lytic phage-host interactions for these genera than DPA. In 17% of the cases there were ambiguous results, which can be further distinguished. There are 5% of the cases in the category “DPA: reduced growth - PKA: lysis” (dark orange), in which the host range of phages was underestimated using the DPA method. Many of these cases were related to jumbo phages. This is due to the fact that members of *Elvirus*, *Pawinskivirus* and *Bruynoghevirus* in particular formed invisible, too tiny or not clearly defined individual plaques with 0.5% top agar. When the lysis behavior is tested with PKA, these phages sometimes achieve even better coverage (43 to 67%) than the other myoviruses (**Figure 2G**). On the other hand, 13% of the cases were classified as “DPA: reduced growth – PKA: no lysis” (light orange). In particular, phages with the morphotype siphovirus accounted for more than 21% in this category, which means that they were probably overestimated by the DPA. If the plaques were cloudy in DPA, often no lysis was observed in the PKA (**Figure 5**). No correlation was found in 6% of the cases with expression “DPA: no lysis - PKA: lysis” and 6% of the cases with the expression “DPA: lysis - PKA: no lysis”. The second case was particularly prevalent in *Litunavirus* and phages with the morphotype siphovirus (**Figure 5**). Since both methods lead to different results and this can have an influence on the selection of phages, both methods must be taken into account for the PST.

**Figure 6:**
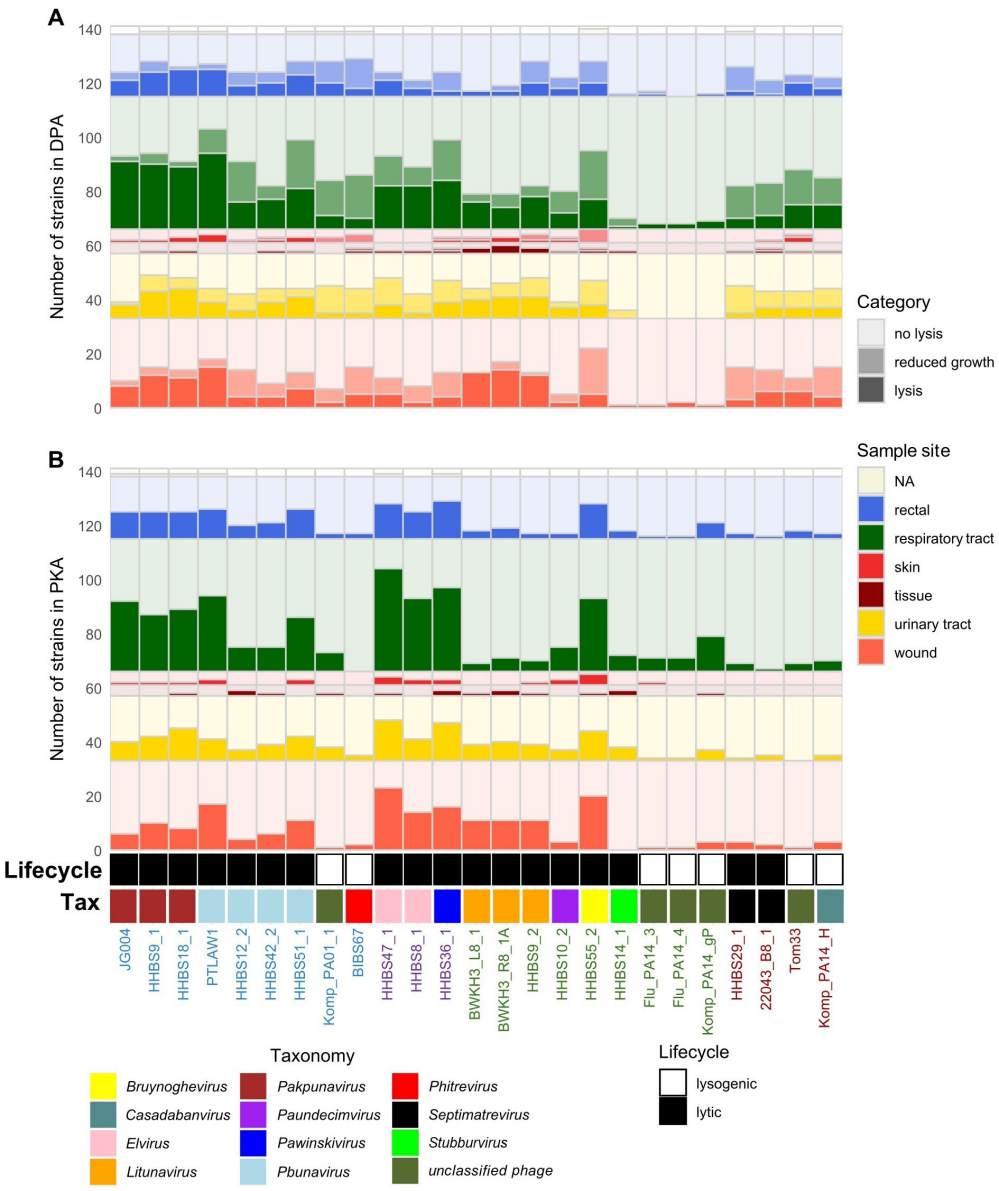
Percentage of lysed clinical *P. aeruginosa* strains depending on the phage and the PST. The phage-host interaction is shown with increasing intensity: DPA assay in three categories (no lysis, reduced growth, lysis) **(A)** and PKA assay in two categories (lysis: normalized AUC < 0.8 and no lysis: normalized AUC ≥ 0.8) **(B)**. Both plots are grouped based on morphotype (colored names at the bottom: myo- (blue) – jumbo phages (violet), podo- (green) or siphovirus (brown)), genus and lifecycle of the phage. The habitats from which *P. aeruginosa* was sampled are light grey = not available, blue = rectal, dark green = respiratory tract, red = skin, brown = tissue, yellow = urinary tract and orange = wound.

This study also investigated whether there are infection-specific phages by analyzing the host spectrum with regard to the isolation type of the hosts. **Figure 6** represents the percentage coverage of the lysed strains depending on the type of infection of strains shown for DPA and PKA. A specificity of certain phages toward *P. aeruginosa* from specific habitats or diseases could not be determined in this study. The only finding was that *Litunavirus* lysed fewer strains from the respiratory tract than myoviruses.

### 3.9 Final ranking of phages suitable for a phage therapy

**Figure 7:**
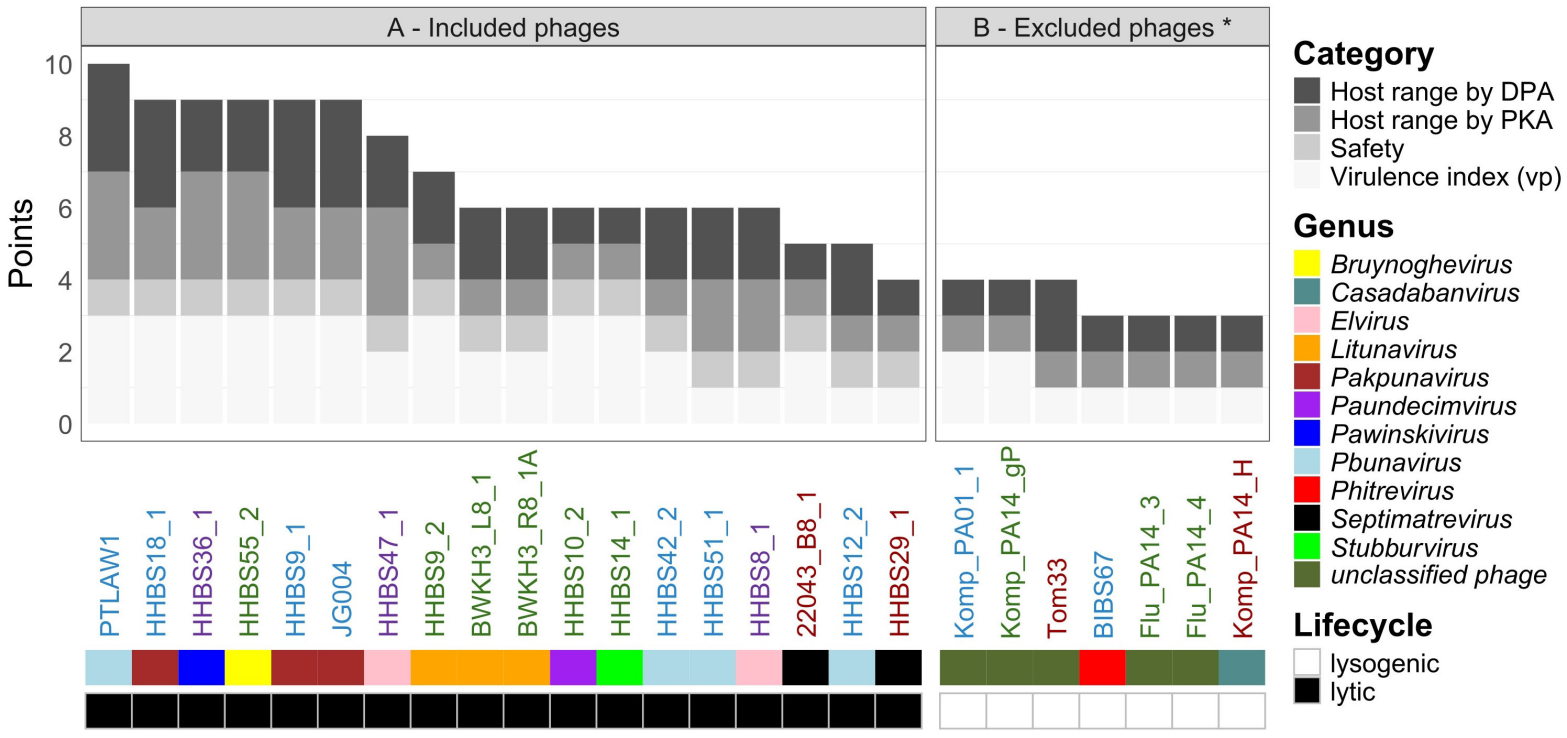
The ranking of phages regarding therapeutic potential and production is visualized by several categories in descending order and subdivided according to safety aspects. Both plots are shown with the morphotype (colored names: myo- (blue) – jumbo phages (violet), podo- (green) or siphovirus (brown)), genus and lifecycle of the phage. **(A)** shows all phages without safety concerns and **(B)** shows all phages with safety concerns due to a lysogenic life cycle *. In both diagrams, the distribution of points is determined as described in **Supplementary Data Sheet 4**. In detail, the points for DPA are as follows: 3 points for ≥ 42 lysed strains, 2 points for 21–42 lysed strains and 1 point for < 21 lysed strains. Classification of host range determined by PKA is: 3 points for ≥ 63 lysed strains, 2 points for 31–63 lysed strains and 1 point for < 31 lysed strains. Strictly lytic phages without toxins, antibiotic resistance genes or genes that induce a lysogenic cycle receive 1 point, otherwise 0 point*. Virulence index (vp) is classified like 3 points: vp ≥ 0.6, 2 points: vp 0.3-0.6 and 1 point: vp < 0.3. In addition, the phages are colored based on their genera, morphotype and lifecycle.

Two aspects need to be distinguished for a ranking of the phage concerning their application in a phage therapy. Which phages are suitable for (individual) therapy and which phages have favorable properties for production. Both aspects should be considered for the selection to ensure an efficient and safe treatment as well as successful production. Here we provide a ranking (**Figure 7**) with multiple factors like safety, host range determined by DPA and PKA and virulence index (vp) included.

We suggest that phages with a broad (*Pakpunavirus*) or narrow (*Stubburvirus*) host spectrum should be assessed equally for therapy if they are effective on a patient isolate. Even more important are the safety aspects. In order to exclude risk factors for the patients, the genomes of the phages were examined. 18/25 phages were strictly lytic, do not encode any virulence factors or antibiotic resistance genes (analyzed with Virulence factor database (VFDB) (Liu et al., 2022) and AMRfinderPlus (Feldgarden et al., 2021) (Altschul et al., 1997; Feldgarden et al., 2021) and could be used for therapy purposes (**Figure 7**). All lysogenic phages (*unclassified phages, Casadabanvirus* and *Phitrevirus*) with integrase, transposase, excisionase or other DNA transposition proteins were excluded (**Figure 7B**) for therapeutic use.

Looking at our ranking, PTLAW1 (*Pbunavirus*) ranked the highest with ten final points. Other phages like HHBS18_1, JG004 and HHBS9_1 (*Pakpunavirus* with morphotype myovirus) received 9 points. They lysed their production host efficiently and fast (**Figure 2H**). HHBS55_2 (*Bruynoghevirus*) and HHBS36_1 (*Pawinskivirus*) were also promising candidates (9 points) because of their great host range in PKA. HHBS47_1 should be preferred (8 points) over HHBS8_1 (both *Elvirus*) (6 points) because of a higher virulence index. Various phages with the podovirus morphotype ranked in the midfield, including the promising phage HHBS9_2 (*Litunavirus*) (7 points). On average, siphoviruses ended up at the bottom of the ranking. The biggest influence was their low virulence index (vp). This made HHBS29_1 (*Septimatrevirus*) difficult to produce in liquid (**Figure 7**, **Supplementary Data Sheet 3**, **Supplementary Figure 2A**)).

### 3.10 Theoretical combination of phages for phage therapy

The effectiveness of phage therapy depends not only on the selection of individual phages but also on a combination of phages. Thus, the theoretical coverage of multiple phage applications was analyzed *in silico* using the Phage Cocktail Optimizer (Abedon, 2020) to identify the phage combination that maximizes coverage. Only safe phages that could be used for treatment were included in this calculation. The maximum coverage of 92% of all strains with at least one phage per bacterium was achieved with six phages, based on the PKA results. For the DPA results, the maximum coverage is 86% with a total of twelve phages combined (**Table 1**). The greatest theoretical coverage was always achieved when phages of different genera were combined. If three phages were theoretically combined in a cocktail, this would result in a coverage of over 74% (DPA) or 86% (PKA) (**Table 1**). Many possible combinations contain PTLAW1 (*Pbunavirus*) and HHBS47_1 (*Elvirus*) or HHBS55_2 (*Bruynoghevirus*).

**Table 1:** Coverage of a theoretical phage combination determined with Phage Cocktail Optimizer (Abedon, 2020) depending on the method and the depth of the cocktail (only safe phages used).

Method	DPA				PKA								
Number of phages in cocktail	2	3	4	12	2	3			4			5	6
Theoretical number of lysed strains	91	104	109	121	113	121			126			129	130
Theoretical percentage of lysed strains	65%	74%	77%	86%	80%	86%			89%			91%	92%
Phage1	HHBS18_1	HHBS47_1	HHBS47_1	**HHBS47_1**	HHBS47_1	HHBS47_1	HHBS47_1	HHBS47_1	HHBS47_1	HHBS47_1	HHBS47_1	HHBS47_1	HHBS47_1
Phage2	PTLAW1	PTLAW1	HHBS36_1	**HHBS8_1**	HHBS55_2	PTLAW1	HHBS36_1	JG004	HHBS36_1	HHBS36_1	JG004	HHBS36_1	HHBS36_1
Phage3		HHBS9_2	PTLAW1	**HHBS9_1**		HHBS55_2	HHBS55_2	HHBS55_2	PTLAW1	HHBS51_1	HHBS36_1	HHBS12_2	HHBS12_2
Phage4			HHBS9_2	**JG004**					HHBS55_2	HHBS55_2	HHBS55_2	HHBS51_1	HHBS51_1
Phage5				**HHBS36_1**								HHBS55_2	HHBS55_2
Phage6				**HHBS12_2**									HHBS10_2
Phage7				**HHBS29_1**									
Phage8				**HHBS14_1**									
Phage9				**BWKH3_L8_1**									
Phage10				HHBS55_2 or PTLAW1									
Phage11				HHBS9_2 or BWKH3_R8_1A									
Phage12				PTLAW1 or HHBS51_1									

*8 combinations of phages possible, only one combination shown. Core backbone of all 8 cocktail combination is displayed in **bold**.

## 4 Discussion

Our data highlights the diversity of *P. aeruginosa* phages. We were able to isolate 25 phages, 18 of which pursue a strictly lytic lifecycle (all isolated from wastewater samples), while only temperate phages could be extracted from the environmental samples (garden compost, tomato, washing machine). New phages should therefore be sequenced at an early stage of the work to determine the life cycle and avoid labor-intensive experiments. All lytic phages could be assigned to previously published genera, but many phages represent a potential new species (**Supplementary Data Sheet 3**). Some temperate phages, on the other hand, were assigned to following new genera: first new genus include Flu_PA14_3, Flu_PA14_4, Komp_PA14_gP, second new genus Komp_PAO1_1 and third genus Tom33.

Our study revealed a high diversity in particular among in the morphotype myoviruses of our *P. aeruginosa* phage panel. Although they all have a hexagonal head and tail, they differ considerably at the genome level. Therefore, it is more contemporary to compare phages within their genus and not their morphotypes (Turner et al., 2021). There are many variations within a phage genus, including insertions, deletions, and mutations within genes. Mutations within the tail proteins are primarily responsible for adsorption to the host (Gaborieau et al., 2024) and the resulting variability of host specificity.

Virus- and plaque morphologies are often used as a basic characterization as they are easy to compare and allow phage identification. The formation of large, clear plaques should be favored over cloudy plaques, as this often represents temperate phages. Small plaques are problematic as well, as they can be difficult to detect. However, phages should only be excluded after sequencing, as morphological characteristics only provide an initial indication of which phages have therapeutic potential. By determining the virulence index (vp), phages can be qualitatively compared for the first time (Storms et al., 2020). For this purpose, MOI-dependent lysis curves were recorded, the AUC was normalized against an uninfected control, and these local virulence indices (vi) are integrated a second time against the log MOI to obtain the virulence index (vp). A comparison of vp of different phages presents some difficulties, as almost all parameters must be identical for vp to be successfully calculated. Normally, only one parameter like growth medium or temperature may be changed in order to achieve optimal comparability. Nevertheless in our data, two parameters (phage and host) were adapted. Our aim was to determine which phage with its host is best suited for production. It has been shown that the production of *Pakpunavirus*, even at low MOIs, results in a significantly greater bacterial reduction than *Litunavirus* (**Supplementary Data Sheet 3**). Some of the phages (HHBS8_1 and HHBS47_1 (*Elvirus*)) lyse only after the onset of the steady-state phase, so that the vp is very small due to the official calculation that includes the time of the steady-state phase of the control. Therefore, for the determination of susceptibility in PKA, we calculated the AUC over 24 h rather than up to the steady-state phase to compensate for the different growth behavior of the strains. We consider that Peters et al, 2023 had similar difficulties in calculating the vp according to the original definition by Storms et al, 2020 and therefore set the cut-off to 8 hours and thus included early regrowth of the bacteria in the virulence index. Normally, phages with a high vp value should be favored. Nevertheless, we would like to raise the question of whether late-lysing phages with different lysis kinetics are even advantageous in combination with other early-lysing phages.

The phages can be differentiated according to their host spectrum. Using our broad phage and host panel, we compared 3525 phage-host interactions with DPA and PKA. We found that the comparability of the two methods strongly depends on the phage genus analyzed (**Supplementary Figure 11**). In particular, members of *Pbunavirus*, *Pakpunavirus* and *Litunavirus* can be compared very well (comparability of the methods on average > 76%), as they form very large plaques. The method DPA has some limitations if the plaque morphology is not unambiguous in the case of small or cloudy plaques. For example, the formation of small plaques of jumbo phages, which lead to the lytic interaction being undetected, could be avoided by decreasing the top agar concentration (Serwer et al., 2007; Yuan and Gao, 2017). However, other problems can then arise, such as the overgrowth of plaques with very mucous clinical isolates, and the plaques sometimes only grow very slowly (HHBS8_1 at 0.3% in **Supplementary Figure 1**) (Staudinger et al., 2014). Low adsorption rates favor the formation of small plaques not only in jumbo phages (Abedon and Culler, 2007). In contrast to the assumption that phages that do not lyse a liquid culture fail to form plaques as published by Haines et al., 2021, our results demonstrate that this behavior is exhibited by siphoviruses in particular. In addition, we were able to show that the plaque morphology of phages with regard to turbidity is dependent on the strain, especially in case of temperate phages. However, the boundaries between clear and cloudy plaques are difficult to distinguish by eye. HHBS55_2 (*Bruynoghevirus*), BWKH3_L8_1 and BWKH3_R8_1A (both *Litunavirus*), which are lytic phages, form cloudy plaques in their original strains (**Figure 2C**) and in some cases also on other strains (**Figure 4**, data not shown).

Considering all these arguments we propose to use PKA as the standard method for PST. It offers several advantages: an already successfully implemented automation, easy performance in replicates and with different MOIs, and quantitative measurements in a high throughput screening format. For most phage-host interactions, PKA is faster, it can detect possible lytic behavior after only a few hours. In addition, several phages can be tested simultaneously or in a combination of phages with antibiotics. However, it should be noted that the lysis behavior can also be caused by toxins, endolysins, tailocins etc. when using crude lysates and therefore purified lysates should be preferred and the results should be validated with those of the DPA (Kim et al., 2020). In the future it may also be possible to predict phage-host interaction *in silico*, as this has already been done for selected bacteria such as *Staphylococcus aureus* (Moller et al., 2021)*, Escherichia coli* (Gaborieau et al., 2024) and *Klebsiella pneumoniae* (Boeckaerts et al., 2024). The prediction tools that are currently available, such as HostPhinder, DeepHost, do not yet differentiate between phage-host interaction in *P. aeruginosa* at the strain level (Villarroel et al., 2016; Ruohan et al., 2022). But regardless of whether DPA and PKA are used as PST, the more important comparison is with the *in vivo* data, so that we can understand which assay better mimics the situation in the patient. So far there is still little data available. In many studies, it would be helpful for the comparison of *P. aeruginosa* phages if both the phage name and the corresponding genus is mentioned.

There is a tendency for phage susceptibility in the classical host spectrum to correlate negatively with antibiotic resistance (**Supplementary Figure 10**). Despite repeated efforts, we were unable to find a matching phage for every clinical *P. aeruginosa* isolate with twelve of 37 known *P. aeruginosa* phage genera. Especially the wound isolates were less covered with the phage panel. To verify possible explanations like anti-phage defense mechanism of the bacteria, interacting prophages or missing receptors, the strains need to be sequenced (Makarova et al., 2020; Markwitz et al, 2022; Georjon and Bernheim, 2023). Since there are limitations regarding the selected phages and antibiotics, further experiments, including *in vitro* studies, need to be performed to verify the correlation.

Our ranking suggests that *Pakpunavirus*, *Pbunavirus*, *Pawinskivirus* and *Elvirus* (myoviruses) as well as *Litunavirus* and *Bruynoghevirus* (podoviruses) have great potential for an application in phage therapy because they lyse many strains and are easy to produce (**Figure 7**). In previous studies, these phage genera have often been used *in vitro* and *in vivo* in animals and humans against *P. aeruginosa* infections (Silva et al., 2022). In particular, various *Pbunavirus* such as Pa193, Pa204, Pb10, which are > 95% identical (coverage x identity) to PTLAW1, have already demonstrated their high potential for phage therapy against *P. aeruginosa* as single phages (**Supplementary Figure 3**) (Forti et al., 2018; Aslam et al., 2019; Alkalay-Oren et al., 2022; Shafigh Kheljan et al., 2023). These phages have also been used in phage cocktails. A metagenomic analysis in 2017 identified eight different phage genera in Pyo Bacteriophage™ (Georgia) against *P. aeruginosa*, which have many similarities with our phages (*Pakpunavirus, Pbunavirus, Phikzvirus, Nankokuvirus, Phikmvvirus* (myoviruses) and *Bruynoghevirus, Litunavirus, unclassified phage* (podoviruses)) (McCallin et al., 2018). For other phage cocktails, such as PP1131 (PhagoBurn) or BX004-A™ (BiomX, Israel), compositions have not yet been reported (Jault et al., 2019; Rappo et al., 2023). However, the use of different genera increases the likelihood that many strains/isolates can be treated (**Figure 5**). Not only do the host ranges of the individual phages often add up, there is also the chance of phage-phage synergy (PPS) (Schmerer et al., 2014). Whether synergistic or antagonistic effects exist for the best predicted cocktails with the specific phages would require further *in vitro* or biofilm analysis. In order to increase the overall coverage, phages with a narrow host range should also be included. Only a few siphoviruses have been used for therapy so far (Silva et al., 2022). We assume that this is due to their narrow host range (**Figures 2F, G**), and also due to the fact that the probability of finding lytic phages with the morphotype siphovirus is much lower, as the majority of *P. aeruginosa* prophages belong to the morphotype siphovirus (Johnson et al., 2022). However, this does not mean that no lytic siphoviruses are found (see 22043_B8_1 and HHBS29_1 (*Septimatrevirus*)).

In addition to safety and the suitable host spectrum, there are other factors that should be included in a ranking. Depending on the indication, efficacy against biofilms could also play a role in the selection. Among others the stability of the phages, which is often tested at different pH values and temperatures. Stability of the phages should be ensured regarding the various therapeutic indications (bladder, lungs, etc.). In addition, phages must be sequenced to exclude possible induced prophages from the host, as *P. aeruginosa* usually has more than one prophage (Johnson et al., 2022). Furthermore, combinations with other phages or antibiotics should be considered for therapy (Nikolic et al., 2022). Phage-antibiotic-antagonism (Gu Liu et al., 2020; Danis-Wlodarczyk et al., 2021a) and phage-phage antagonism should be avoided (Schmerer et al., 2014). At the same time, it should be tested whether phages of the same genus should be used together, as they probably have similar receptors (Pleteneva et al., 2008). In the best case the phages would be selected and combined in a way that it would make it difficult for the bacteria to adapt via the receptor or defense systems.

## 5 Conclusion

25 *P. aeruginosa* phages were characterized and compared showning a great diversity within all morphotypes. A combination of six phages could theoretically lyse 92% of clinical *P. aeruginosa* strains. Further, we showed that especially *Pakpunavirus*, *Pbunavirus, Pawinskivirus, Elvirus* (all myoviruses), *Litunavirus* and *Bruynoghevirus* (all podoviruses) showed the greatest potential in the proposed ranking and should be employed with priority, as they have already been used for phage therapy. Siphoviruses were less suitable. Phages with a lysogenic cycle also have less potential due to their narrow host range and efficiency (low vp value), as well as safety concerns regarding phage therapy.

We also compared the classic DPA (0.5% top agar) with the liquid PKA (MOI 0.1). Both methods are easy to perform but offer different limitations depending on the phage. Host analysis using DPA did not detect phages that form very small plaques, while PKA could not clearly state whether lysis by some siphoviruses and temperate phages was caused by phage activity. Overall, we suggest that PKA is the better method for phage susceptibly prediction because it is usually faster and less prone to individual interpretation. However, both methods should also be compared with *in vivo* data from patients in the future.

## Data Availability

The datasets presented in this study can be found in online repositories. The names of the repository/repositories and accession number(s) can be found in the article/**Supplementary Material**.
