# Correction: Systematic bacteriophage selection for the lysis of multiple *Pseudomonas aeruginosa* strains

**DOI:** 10.3389/fcimb.2025.1650832

**Published:** 2025-08-20

**Authors:** Finja Rieper, Johannes Wittmann, Boyke Bunk, Cathrin Spröer, Melanie Häfner, Christian Willy, Mathias Müsken, Holger Ziehr, Imke H.E. Korf, Dieter Jahn

**Affiliations:** ^1^ Pharmaceutical Biotechnology, Fraunhofer Institute for Toxicology and Experimental Medicine (ITEM), Braunschweig, Germany; ^2^ Institute of Microbiology, Braunschweig University of Technology, Braunschweig, Germany; ^3^ Leibniz Institute DSMZ-German Collection of Microorganisms and Cell Cultures GmbH (DSMZ), Braunschweig, Germany; ^4^ Department Trauma & Orthopedic Surgery, Septic & Reconstructive Surgery, Research and Treatment Center Septic Defect Wounds, Federal Armed Forces of Germany, Military Academic Hospital Berlin, Berlin, Germany; ^5^ Central Facility for Microscopy, Helmholtz Centre for Infection Research (HZI), Braunschweig, Germany; ^6^ Institute of Microbiology, Braunschweig Center of Systems Biology (BRICS), Braunschweig, Germany

**Keywords:** bacteriophages, *Pseudomonas aeruginosa*, phage susceptibility testing, antibiotic resistance, phage selection

The **Conflict of interest statement** was erroneously given as “Author XY was employed by Leibniz Institute DSMZ, which is an independent, non-profit research infrastructure.

All authors declare that the research was conducted in the absence of any commercial or financial relationships that could be construed as a potential conflict of interest.”

The correct **Conflict of interest statement** is “Author BB, CS alnd JW was employed by Leibniz Institute DSMZ, which is an independent, non-profit research infrastructure.

The remaining authors declare that the research was conducted in the absence of any commercial or financial relationships that could be construed as a potential conflict of interest.”

The original version of this article has been updated.

In the published article, there was a mistake in the numbers of isolated phages. The number of myoviruses was given as “13” and the number of podoviruses as “eight”.

A correction has been made to the section 3.2 Phage morphology, Line 284:285:

“Using transmission electron microscopy (TEM), the 25 phages were classified as twelve myoviruses including three jumbo phages, four siphoviruses and nine podoviruses (Figure 1)”.

The original version of this article has been updated.

